# Functional outcome of unstable pelvic fractures treated in a level III hospital in a developing country: a 10-year prospective observational study

**DOI:** 10.1186/s13018-022-03088-3

**Published:** 2022-04-04

**Authors:** Chunteng Theophile Nana, M. A. Ngo-Yamben, Pius Fokam, Ali Mahamat, F. M. Bombah, M. Ekani Boukar, Muluem Kenedy, A. Chichom-Mefire

**Affiliations:** 1 Department of Surgery, Faculty of Health Sciences, University of Buea, Buea, Cameroon; 2 Department of Surgery and Specialties, Faculty of Medicine and Biomedical Sciences, University of Yaounde 1, Yaounde, Cameroon; 3 Department of Surgery and Specialties, Faculty of Medicine and Pharmaceutical Sciences, University of Douala, Douala, Cameroon

**Keywords:** Functional outcome, Unstable, Pelvic fracture, Cameroon

## Abstract

**Background:**

Unstable pelvic fractures are severe and life-threatening injuries with high morbi-mortality rates. Management of these fractures is a major challenge in orthopaedic practice in limited resource communities. The aim of this study is to evaluate the functional outcome of unstable pelvic fractures managed in a hospital with limited diagnostic and therapeutic facilities.

**Methodology:**

This was a hospital-based prospective observational study carried out from 1st of January 2009 to 31st of December 2018 at the Limbe Regional Hospital, a level III health institution in the South-West region of Cameroon.

**Results:**

A total of 68 patients were included in the study. The ages ranged from 18 to 80 years with a mean age of 39 ± 5 years. The average follow-up duration at the latest visit was 36 months (range 3–84 months). There were 59 cases that were evaluated. The overall average Majeed score was fair. Poor outcomes were noted in patients aged 60 years and above, those with co-morbidities, and those managed conservatively.

**Conclusion:**

Although the functional outcomes following unstable pelvic fractures have improved with modernised diagnostic and therapeutic modalities, it is not the case in poor resource settings where the lack of these modalities makes the management challenging, consequently affecting the functional outcome.

## Introduction

Unstable pelvic fractures are severe and life-threatening orthopaedic injuries with an estimated mortality rate of up to 19–31% [1]. This high mortality is due to the fact that these fractures occur usually as a result of high energy trauma, and most of the injured victims are cases of polytrauma, presenting with associated injuries to other body parts (head, chest, abdomen, extremities) and haemodynamic instability due to haemorrhage resulting from disruption of pelvic venous and arterial vessels [2]. Haemodynamic instability associated with the pelvic fractures increases the mortality rate to 20–50% [3].

Management of unstable pelvic fractures still remains one of the major challenges in orthopaedic practice in limited resource communities where the burden of injury is on the rise [4, 5]. Most patients present with severe associated injuries that are difficult to treat with the diagnostic and therapeutic means available in the local facilities [6]. Injured victims with unstable pelvic fractures are best initially managed by a multidisciplinary trauma team that should include a trauma surgeon, orthopaedic surgeon, radiologist with interventional radiology abilities, urologist and a neurosurgeon [7]. This multidisciplinary team is lacking or not well organised when available in most hospital settings in low- and low-middle-income countries [4, 6, 8].

A large proportion of patients with pelvic fractures who survive their injuries may incur temporal or permanent disabilities affecting function [9]. The management of these disabilities and complications usually require somewhat exorbitant expenditures that will consequently place heavy socio-economic burdens on the injured individuals, their families, and the society as a whole [10, 11]. There is scarce literature describing the functional outcome of unstable pelvic fractures treated in a resource-limited setting. The aim of this study is to evaluate the functional outcome of unstable pelvic fractures and identify the factors affecting them, in a context where there are limited diagnostic and therapeutic facilities.

## Patients and methods

### Study design

This was a hospital-based prospective observational study carried out from 1st of January 2009 to 31st of December 2018 at the Limbe Regional Hospital. It is a level III health institution located in the South-West region of Cameroon and has a capacity of 200 beds. The surgical ward has a capacity of 26 beds, and there are five surgical specialists including one orthopaedic surgeon, one general surgeon, one urologist, and two ENT surgeons.

### Study population and sampling

The study involved all patients with unstable pelvic fractures who were admitted and treated at the Limbe Regional Hospital within the 10-year period. The sampling was done in a consecutive manner.

### Selection criteria

Inclusion criteria: All cases of unstable pelvic fractures aged above 18 that were admitted, treated, and followed-up at the Limbe Regional Hospital were included in the study.

Exclusion criteria: Patients from whom relevant data could not be obtained and those who did not consent for the study were excluded. Patients who were initially treated for the same fracture in another hospital were equally excluded.

### Study procedure

Patients with unstable pelvic fractures who were admitted, treated, and discharged were approached during the routine visits at the clinic or contacted by telephone to report for evaluations. Those who met the inclusion criteria were counselled on the aim and importance of the study. Information concerning patient demographics, history, clinical evaluation, and radiological examinations were recorded on a data entry form.

On admission, all patients were initially evaluated at the emergency department of the hospital. Patients with unstable clinical states were urgently resuscitated appropriately. All those who were suspected of having a pelvic fracture had a pelvic binder applied at the emergency, or were taken to the operating room where external fixators or pelvic clamps were applied. After adequate stabilisation, definitive pelvic fixation was planned for the patients.

In this study, pelvic fractures were classified according to the Tile’s classification system. Tile B and Tile C are classified as unstable while Tile A injuries are considered stable [12].

The patients were definitively managed either surgically or conservatively. The decision for definitive management modality was guided by patients’ age, co-morbidities, clinical condition, injury pattern, and financial constraints. Conservative management modalities included the use of pelvic binders, pelvic clamps, and skeletal traction depending on the fracture type and pattern. Surgical stabilisation modalities included internal fixations with plates and screws, external fixations, ilio-sacral screws or combination of these, without the use of image intensifier. The patients were all asked not to fully bear weight until after 3 months or when there’s radiological evidence of consolidation. After discharge, follow-up visits were scheduled every 45 days during which the patients were thoroughly examined for any complications. The functional outcome was evaluated using the Majeed scoring system [13]. This score includes constitutes four grades determined clinically and evaluated on 100 points. The outcome is graded as excellent (value > 85), good (value between 70 and 84), fair (value between 55 and 69), and poor (value < 55).

### Data management and analysis

Data were kept secured and all information was recorded in a computer protected by a password. Epi Info software was used to analyse these data. Results were represented on tables to ease organisation and comprehension.

### Ethical consideration

Ethical clearance was obtained from the Faculty of Health Sciences of the University of Buea Institutional Review Board (IRB). An administrative approval was obtained from the regional delegate of public health and the director of the Limbe Regional Hospital. A consent form was presented to participants explaining the procedure and goals of the research.

## Results

### General characteristics of the study population

In this study, 77 patients were managed for an unstable pelvic fracture. Nine who did not fulfil the inclusion criteria were excluded from the study. A total of 68 patients were included in the study. The ages of the patients ranged from 18 to 80 years with a mean age of 39 ± 5 years. The age group of 20 – 40 years was the most represented (58.88%, *n* = 40). There were more males than females giving a male-to-female ratio of 2:1 (Table 1). Tile B injuries were more common (66.17%, *n* = 45) than Tile C (33.82%, *n* = 23). A total of 15 cases were open fractures while the rest were closed fractures. Road traffic accidents accounted for the majority of injuries (73.53%, *n* = 50). Concerning the initial clinical states of the cases on admission, 23 (33.82%) presented with hypovolemic shock, 50 (73.52%) had Injury Severity Scores (ISS) of more than 9, and only 2 (2.94%) had Glasgow Coma Scores less than 13. Associated injuries were found in 30 cases (44.12%,), the most common being fractures of the extremities. Co-morbidities were found in 21 cases (30.88%) and included osteoporosis (6), malignancies (3), hypertension (6), and diabetes mellitus (6). Non-operative management was done in 13 cases (19.11%), while 55 (80.89%) were managed surgically. Duration of hospital stay varied from 21 to 120 days with a mean of 36.54 ± 17 days. Complications were recorded in 46 cases (67.65%), the most common being pressure ulcers (15 cases). Other complications were surgical site infections (13 cases), severe anaemia (10 cases), thromboembolic events (6 cases), and urinary tract infections (2 cases).

**Table 1:** General characteristics of unstable pelvic fractures

Variables	Items	Number
Age	Less than 20	5
	20–40	40
	41–60	15
	61–80	8
Gender	Males	45
	Females	23
Types of unstable pelvic fracture	Tile B	45
	Tile C	23
	Open fracture	15
	Closed fracture	53
Mechanism of injury	Road traffic crash	50
	Falls	8
	Crush (other than road traffic crash)	5
	Assault	2
	Direct impact from falling objects (walls, trees, rocks)	3
Clinical states on admission and associated lesions	Hypovolemic shock	23
	Glasgow coma score lees than 13	2
	Injury severity score more than 9	50
	Upper and lower limb fractures	13
	Chest injuries	3
	Abdominal injuries	4
	Urogenital injuries	8
	Head injuries	2
	Neurological injuries	3

### Prospective review at the clinic

The average follow-up duration at the latest visit was 36 months (range 3–84 months). There were 59 cases that were evaluated. The overall average Majeed score was 67.81 on a scale of 100, representing a fair score as shown in Table 2. Concerning the various score categories, six were excellent, 19 were good, 26 were fair, and 8 were poor as shown in Table 3. Associations of various factors with the Majeed score were investigated to determine their significance with respect to the functional outcome of the 59 cases (Table 4). Poor outcomes were noted in patients aged 60 years and above (average score 46.00, *p* < 0.001), those with co-morbidities (osteoporosis, malignancies and diabetes mellitus), and those managed conservatively (average score 53.08, *p* < 0.001). Female patients had better outcome than males but the difference was not statistically significant (*p* < 0.553). Good average outcome scores were noted in patients less than 30 years old, and in those managed surgically. There were no significant associations between functional outcome and the presence of associated injuries, mechanisms of injury, and length of hospital stay.

**Table 2:** Functional outcome according to the Majeed scale

Elements of the Majeed score	Average score	Range
Pain/30	24.58	5–28
Work/20	15.65	5–20
Sitting/10	6.45	2–8
Sexual intercourse/4	2.68	0–4
Walking aids/12	9.24	6–10
Walking distance/12	9.21	5–11
Range and total	67.81 ± 12.2	40–90

**Table 3:** Categories of functional outcome

Majeed score ranges	Number	Percentage
Less than 55 (poor)	8	13.55
55–69 (fair)	26	44.07
70–84 (good)	19	32.20
> 85 (excellent)	6	10.17

**Table 4:** Associations of various factors with the Majeed score

Variables	Mean Majeed score ± (standard deviation)	p-value
*Gender*		
Male	67.11 (11.600)	0.553
Female	69.00 (13.534)	
*Age categorised*		
< 30	81.30 (11.186)	< 0.001
30–60	68.10 (8.142)	
60 +	46.00 (7.550)	
*Type of fractures*		
Tile B	68.64 (12.886)	0.422
Tile C	66.09 (10.937)	
Open fracture	69.27 (9.801)	0.592
Closed fracture	67.33 (12.896)	
*Management modality*		
Surgical	70.96 (9.913)	< 0.001
Conservative	53.08 (11.341)	
*Co-morbidities*		
Osteoporosis	42.83 (4.916)	< 0.001
Malignancy	41.67 (2.887)	< 0.001
Diabetes	52.75 (14.607)	< 0.001
*Associated injuries*		
Extremity fractures	65.68 (13.830)	0.792
Chest injury	69.06 (12.326)	
Abdominal injury	69.60 (7.777)	
Urogenital injury	59.33 (13.151)	

## Discussion

Unstable pelvic fractures are relatively rare but severe life-threatening injuries associated with high morbidity and mortality rates [1]. The treatment of such complex injuries is challenging in situations where human resources, diagnostic, and therapeutic facilities are limited or less developed. The purpose of this study was to evaluate the functional outcomes of patients with unstable pelvic fractures managed in a level III hospital in the South-West region of Cameroon.

Majority of the patients had fair functional outcome scores according to the Majeed scale. Consequently, the overall average functional outcome was evaluated to be generally fair. The overall functional outcomes were good to excellent in studies in other low-middle countries [14, 15] and developed countries [16–18]. The better outcomes reported by other authors are probably due to available resources and specialists present in their various hospitals which were mostly tertiary. It is recommended to best manage unstable pelvic fractures by a multidisciplinary trauma team in order to optimise short- and long-term outcomes [7]. In addition to the limited diagnostic and therapeutic facilities, the recommended multidisciplinary team is lacking in our level III hospital, and hence the fair outcome scores observed.

Although altogether fair, the functional outcome was slightly better in females than males but the difference was not statistically significant. This could be explained by the fact that the females could have been involved in less violent mechanisms of injury. Patients aged 60 years and above had poor outcomes as compared to the good and fair outcomes in patients less than 30 years and those aged between 30 and 60 years, respectively. Moreover, the presence of co-morbidities was associated with poor functional outcome. No co-morbidities were recorded in patients less than 30 years old. This results are similar to findings in other studies in which young patients without co-morbidities had better outcome [18–20]. Elderly patients with pelvic ring fractures in general have been found to have serious consequences, poor functional outcome and a poor quality of life following their injuries [21, 22]. In this study, there was no statistical difference in outcomes as concerns the type of fracture (open or closed) and the pattern of fracture (Tile B and Tile C). Conversely, open pelvic fractures are associated with severe infections, neurologic injuries, and pelvic floor dysfunction which subsequently may lead to chronic fistula, intercourse pain, and erectile dysfunction which all contribute to the poorer functional outcomes [23–25].

Associated injuries were common in this study and included extremity fractures, chest injuries, abdominal injuries, and urogenital injuries. Associated injuries have been reported to negatively affect the functional outcome of unstable pelvic fractures despite adequate radiographic prof of surgical reduction [26]. Although concomitant injuries were not associated with poor functional scores, the presence of associated injuries has possibly contributed to increase morbidity in our study because neither good nor excellent average scores were recorded. Patients with urogenital injuries had the lowest average scores, but the difference was not statistically significant.

Patients with unstable pelvic fractures who were managed conservatively had a poor average outcome while those who were managed surgically fared better. The reasons for conservative management in this study include financial constraints, unstable clinical conditions, presence of severe associated injuries, and advanced age. The poor functional outcome observed might be attributed to the prolonged immobilisation with its associated risks (bed sores, urinary tract infections), and co-morbidities. Our local facility could not successfully treat some patients who sustained multiple associated injuries, complex urogenital injuries with/without neurological damage. These ones were referred to more specialised centres for treatment but majority declined due to lack of necessary finances. Surgical treatment is the recommended treatment modality for unstable pelvic ring fractures. It is associated with satisfactory functional outcome and provides anatomical reduction of the pelvic ring [23–27].

The Majeed score used in this study to evaluate functional outcome does not capture neurological impairments, which have relevant prognostic influence in patients with unstable pelvic fractures [13]. However, there were 3 cases of neurological injuries that consisted of 2 cases of sciatic nerve neurapraxia, and a case of open fracture with probable sacral plexus injury (manifesting as rectal and urinary incontinence). All the cases recovered fully as was confirmed during routine evaluations at the clinic. Neurological injuries are common in unstable pelvic fractures, and most cases are due to disruption of the lumbosacral plexus during the time of injury or due to iatrogenic injuries from surgery and traction [27–29].

This is probably the first study that prospectively evaluates the functional outcome of unstable pelvic fractures managed in a level III hospital with limited facility and manpower, a common setback faced by many poor resource settings especially in sub-Saharan Africa [6, 8].

## Conclusion

Unstable pelvic fractures are considered as a less common subset of traumatic injuries. The early consequences are usually fatal. Long-term complications are common and have a direct impact on the patients’ functions. The functional outcomes following such injuries have improved with modernised diagnostic and therapeutic modalities. Nevertheless, it is not the case in poor resource settings where the lack of these modalities makes the management challenging, and consequently affects the functional outcome. The overall functional outcome in this study was fair. Poor outcomes were generally observed in elderly patients, those with co-morbidities, and those who were managed conservatively.

## Limitation

Although this study evaluates functional outcomes of unstable pelvic fractures managed in resource-limited setting and some details about associated injuries, the prospective nature does not give the prevalence of unstable pelvic ring injuries and the actual acute clinical states of the patients after injury.
